# Heterogeneity in retinoblastoma: a tale of molecules and models

**DOI:** 10.1186/s40169-017-0173-2

**Published:** 2017-11-09

**Authors:** Sonya Stenfelt, Maria K. E. Blixt, Charlotta All-Ericsson, Finn Hallböök, Henrik Boije

**Affiliations:** 1 Department of Neuroscience, Uppsala University, 75124 Uppsala, Sweden; 2 S:t Eriks ögonsjukhus, Karolinska Institutet, Stockholm, Sweden

**Keywords:** Cancer, Cell-of-origin, Genetics, Horizontal cells, *MYCN*, Photoreceptors, *OTX2*, *RB1*

## Abstract

Retinoblastoma, an intraocular pediatric cancer, develops in the embryonic retina following biallelic loss of *RB1*. However, there is a wide range of genetic and epigenetic changes that can affect *RB1* resulting in different clinical outcomes. In addition, other transformations, such as MYCN amplification, generate particularly aggressive tumors, which may or may not be *RB1* independent. Recognizing the cellular characteristics required for tumor development, by identifying the elusive cell-of-origin for retinoblastoma, would help us understand the development of these tumors. In this review we summarize the heterogeneity reported in retinoblastoma on a molecular, cellular and tissue level. We also discuss the challenging heterogeneity in current retinoblastoma models and suggest future platforms that could contribute to improved understanding of tumor initiation, progression and metastasis in retinoblastoma, which may ultimately lead to more patient-specific treatments.

## Introduction

The term heterogeneity in cancer can be used to describe the diversity in disease behavior between different patients and within a tumor in a single patient. This intrapatient and intratumoral heterogeneity complicates cancer treatment and patient prognoses stands to benefit from deeper understanding of these differences on a molecular, cellular and tissue level [1].

Heterogeneity is also a key element in childhood cancers. Pediatric tumors generally arise after fewer genetic alterations than those in adult patients [1]. Yet, neoplastic growth appears at different times in different patients, displaying both intrapatient and intratumoral disparity in marker expression, making it difficult to identify the cell-of-origin for these tumors. The mechanism for this heterogeneity has intrigued researchers for over two centuries and put retinoblastoma, a rare pediatric cancer of the developing retina, in the spotlight [2]. Retinoblastoma accounts for only ~ 3% of all childhood cancers, has a worldwide incidence of approximately 1 in 20,000 live births, and typically occurs in children younger than 5 years. This cancer was first described in 1809 by the Scottish surgeon James Wardrop, as white brain-like intraocular tumors of retinal origin. Wardrop predicted that the only life-saving option for these patients was enucleation, surgical removal of the eye [3]. The study of retinoblastoma in patients, cell lines and animal models has not only generated better diagnosis and treatment strategies, but also fostered numerous discoveries regarding the basic principles of cancer [4, 5]. The aim of this review is to summarize the heterogeneity present in retinoblastoma at a genetic, cellular and tumor level, and relate this to possible future patient-specific therapies. We will also discuss how different research models may impact experimental results, explain some of the observed heterogeneity, and provide valuable insights into tumor initiation and progression.

## Genetic heterogeneity

Retinoblastoma tumors can be hereditary or spontaneous and they display a high degree of genetic heterogeneity. In 1971 Knudson put forward his two-hit hypothesis, postulating that two rate-limiting mutational events in the retinoblastoma susceptibility gene (*RB1)* were required for tumor formation in retinoblastoma [6]. *RB1* plays a central role in cell cycle regulation and its loss makes cells sensitive to neoplastic transformation. Numerous mutations have been described in the *RB1* gene but several other genes have also been implicated to play a role in retinoblastoma.

### RB1 mutations

As previously mentioned, most retinoblastoma cases are caused by a biallelic loss of function of the tumor suppressor gene *RB1*. Only 1% of children who carry an *RB1* mutation remain unaffected [7]. In the familial, hereditary, form of retinoblastoma (~ 40% of all cases) the first mutational hit occurs in the germline, generating an *RB1* ^+/−^ genotype, and the second hit occurs sporadically in the somatic cells. The hereditary *RB1* mutation is transmitted in an autosomal-dominant fashion [8]. In the non-familial, sporadic retinoblastoma (~ 60% of all cases), two sporadic mutational hits occur in the somatic cells.

**Fig. 1:**
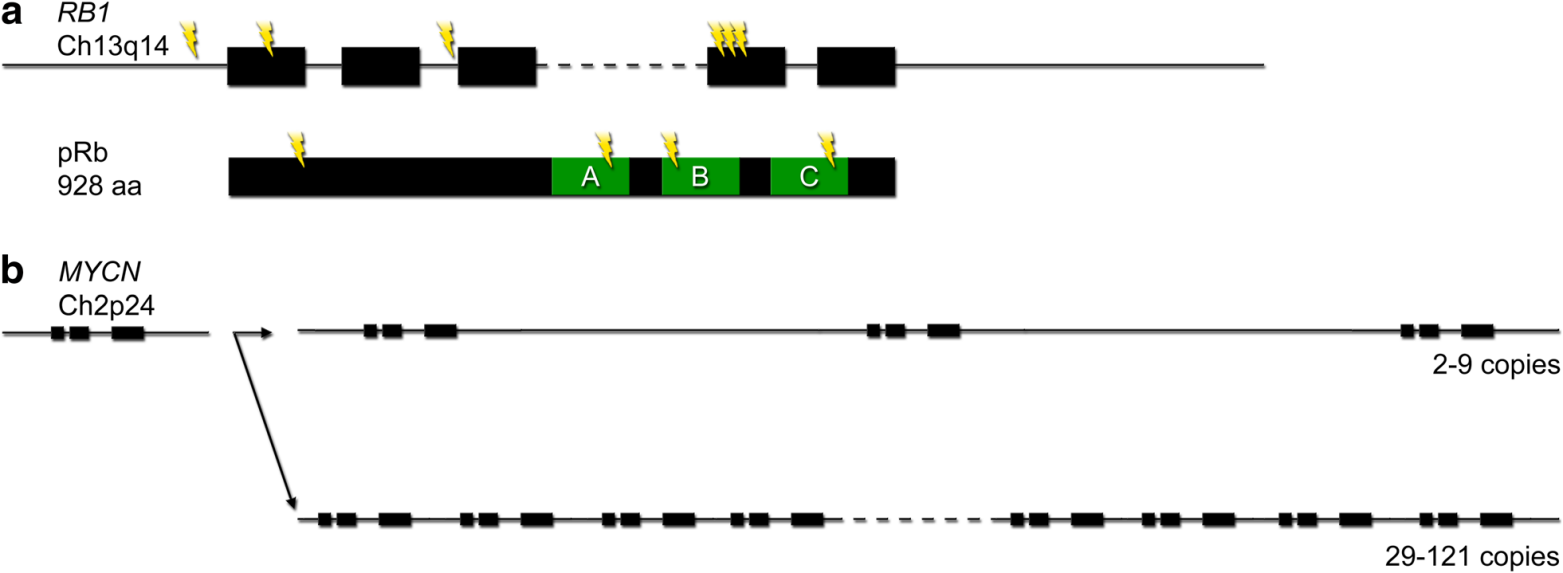
Genetic variants in *RB1* and *MYCN*. **a** Mutations in *RB1* have been found in the promoter region, affecting transcription, in coding regions, introducing premature stop-codons, and in introns, affecting splicing. Hot-spots do exist but only make up ~ 40% of all known mutations. Besides the introduction of premature stop-codons there are also three binding regions where amino acid substitutions can affect the binding ability. As these domains interact with different factors one may end up with a situation where proliferation is affected but not apoptosis, thereby affecting the penetrance and severity of retinoblastoma. **b** *MYCN* amplification is a common trait of retinoblastoma. Here, the difference in the size of the region duplicated, and the number of copies, affect tumor progression

The *RB1* tumor suppressor gene encodes for the pRb protein that regulates transcription of cell cycle genes. pRb mainly acts by interacting with the E2F family of transcription factors, thereby restricting expression of genes required for cell proliferation [9]. For extensive reviews regarding pRb and the cell cycle see [10, 11]. The *RB1* gene can have a variety of mutations such as point mutations, small indels, large deletions, duplications, or even mutations in the regions that regulate transcription [12] (Fig. 1a). It has also been reported that chromothripsis, a massive genomic rearrangement in a single catastrophic event, at the *RB1* locus may cause gene inactivation [13]. Epigenetic changes can also contribute to tumor progression where hypermethylation of promoter regions affect transcription [14, 15].

The *RB1* gene is located on chromosome 13q14, spans 180 kb, and has 27 exons. There is no complete 3D structure for the 928 aa large protein but several binding pockets have been described (Fig. 1a). Exons 13–21 encode for one such binding site and many mutations causing retinoblastoma are found in this pocket [16]. Although mutation hot-spots have been identified they only account for some 40% of the cases. A recent study described the distribution of *RB1* mutations in 1173 patients and classified them as; nonsense (37%), frameshift (20%), splice (21%), large indel (9%), missense (5%), chromosomal deletions (7%), and promoter (1%) (see Fig. 2 in [17] for distribution of germline mutations across the RB1 gene).

**Fig. 2:**
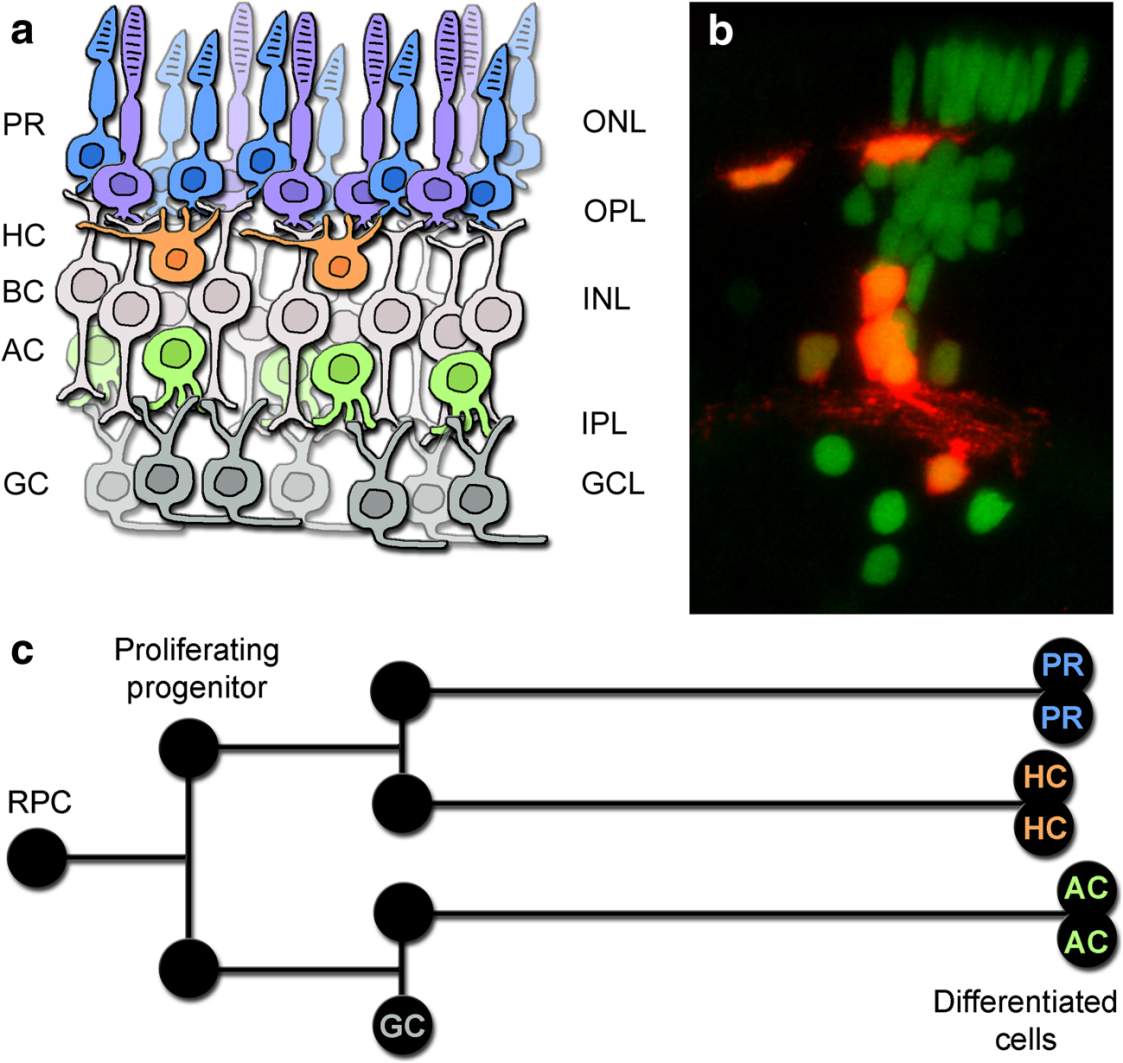
Retinal architecture and cellular relationships. **a** The retina is a laminar structure where the cell bodies of cone and rod photoreceptors (PRs) are located in the outer nuclear layer (ONL), horizontal cells (HCs), bipolar cells (BCs), and amacrine cells (ACs) are located in the inner nuclear layer (INL), and ganglion cells (GCs) are situated in the ganglion cell layer (GCL). These nuclear layers are interspersed by the outer and inner plexiform layers (OPL/IPL) containing the neural connections. **b** Transplantation strategies in zebrafish allow visualization of the clonal expansion performed by a single retinal progenitor cell (RPC) of the optic cup. Here, H2B-GFP is used to label all cells while Ptf1a-dsRed marks horizontal and amacrine cells [43]. These results demonstrate the multipotency of RPCs and highlight the close relationship between some cell types. **c** Additional studies have shown that horizontal cells and photoreceptors often share a common progenitor. The lineage also visualizes the different types of cells where retinoblastoma may arise

### Additional genetic modifications

Human cancers are known to have elaborate mechanistic strategies to evade apoptosis, promote limitless replication, tissue invasion and insensitivity to anti-growth signals [18]. Although mutations in *RB1* are often considered a prerequisite for retinoblastoma initiation, further genomic changes may drive malignancy by activating oncogenes and inactivating tumor suppressor genes [19]. Previous studies on tumor samples have identified recurrent genomic gains at 1q32, 2p24, 6p22 and losses at 16q22 [20]. The biallelic loss of *RB1* has been suggested to cause retinomas, benign non-proliferative retinal tumors. However, data suggests that the transition into retinoblastoma requires further copy number changes in key genes—gain in *KIF14*, *E2F3*, *MYCN* and loss in *DEK* and *CDH1* [13, 20–25]. Other copy number changes reported in retinoblastoma include gains of *DDX1*, *MDM4* and *OTX2*, and loss of *BCOR* and *RBL2* [13, 24–26].

### RB1^+/−^ and RB1^+/+^ retinoblastomas

Different approaches can be used to detect around 95% of the *RB1* mutations; DNA and RNA sequencing to identify mutations and splice mutations, MLPA (Multiplex ligation-dependent probe amplification) and karyotype or chromosomal microarrays to identify chromosomal rearrangements, and promoter hypermethylation to detect gene silencing [27]. However Rushlow et al. reported tumors with only one mutation in *RB1* (RB1^+*/*−^) and even cases with two functional alleles (RB1^+*/*+^) [22]. There is an on-going debate on whether tumorigenesis can be driven without *RB1* loss, for example via a single oncogenic lesion, such as amplification of *MYCN* [13, 22]. The proto-oncogene *MYCN* acts as a transcription factor, promoting proliferation by controlling the expression of certain cell cycle genes [28]. In neuroblastoma *MYCN* amplification (MYCN^A^) is associated with the classical hallmarks of tumor progression and metastasis, such as adhesion, motility and tissue invasion [29]. MYCN^A^ has been found in retinoblastomas both with mutated or non-mutated *RB1* genes [13, 22]. In a study by Kooi et al. it was shown that focal high-level *MYCN* amplification and *RB1* germline mutations were associated with early diagnosis, and that homozygous *RB1* loss, chromotripsis of chromosome 13, and alterations of chromosomal arms were associated with late diagnosis [30].

In a cohort of 1068 retinoblastoma patients, Gallie and co-workers identified a subset of 29 patients (2.7%) with no detectable mutation in *RB1*, where 52% of these had MYCN^A^. The RB1^+/+^MYCN^A^ tumors were described as unilateral with distinct histology, earlier onset, and more aggressive behavior than RB1^−/−^ tumors [22]. In another study, Dyer and co-workers implied that identifying retinoblastomas driven exclusively by *MYCN* amplification is challenging since it requires excluding all other mechanisms of *RB1* gene inactivation including somatic nucleotide variations, indels, loss of heterozygosity, deletions, translocations and promoter hypermethylation [13]. In mice, *MYCN* overexpression alone was insufficient to drive retinoblastoma development, however, tumors formed when overexpression was combined with *RB1* deletion [31]. In another recent publication three retinoblastoma subtypes were compared in human tumors based on *RB1* and *MYCN*; RB1^−/−^MYCN^A^, RB1^+/−^MYCN^A^, and RB1^+/+^MYCN^A^ [32]. The RB1^+/−^MYCN^A^ subtype is particularly interesting as this genotype, according to Knudson’s two-hit hypothesis, would not give rise to tumors. The explanation provided by Ewens et al. is that the effect of the *RB1* gene can be repressed on the protein level via phosphorylation of pRb. The authors argue that the loss of the tumor suppressor function is central for the initiation of retinoblastoma tumorigenesis and that it is independent of MYCN^A^ [32]. Unlike earlier observations of lower frequency of secondary copy number changes in RB1^+/+^MYCN^A^ tumors [22], Ewens et al. found no significant difference in the number of copies of *KIF14*, *DEK*, and *CDH11* between MYCN^A^ and *MYCN*-low tumors. There was however a significant difference in copy number gains in *E2F3* where a smaller fraction of MYCN^A^-tumors showed a gain (21%) compared to *MYCN*-low (58%) tumors [32]. Interestingly, there is a difference in the amplification of *MYCN* where classic RB1^−/−^ tumors have 2-9 copies of *MYCN* while RB1^+/+^ tumors displayed 28-121 copies, with a much narrower amplicon [22] (Fig. 1b).

The genetic alterations following *RB1* loss is highly variable and this heterogeneity affect tumor progression and patient outcome. Combined, these studies reveal the genetic and epigenetic complexity of retinoblastoma. Considering the difference in phenotype between RB1^+/+^ and RB1^+/+^MYCN^A^ genotypes, it is possible that it may be a question of different tumors combined under the name “retinoblastoma”. Detailed knowledge regarding the mutations allows a more accurate prognosis and a better tailored treatment of patients.

## Cellular heterogeneity and the proposed cell-of-origin for retinoblastoma

One important aspect when studying retinoblastoma has been to try to establish the cell-of-origin. It is puzzling why children with heritable *RB1* mutations develop tumors in the retina but no other types of cancer at a young age. Childhood tumors are thought to arise after fewer mutations indicating the presence of tumor-like properties within the cell-of-origin. Retinal cells may have a different sensitivity to chromosomal instability, which affects the apoptotic pathways. Identifying the cell-of-origin, studying the developmental programs and cellular context, may help us to understand why certain cells undergo neoplastic transformation as a result of *RB1* inactivation. The origins of developmental tumors are also more difficult to study as the internal and external environment constantly changes, affecting epigenetic features, cellular competence and cell cycle regulation.

### Cell-of-origin

The neural retina is comprised of five main neural cell types; rod and cone photoreceptors, horizontal cells, bipolar cells, amacrine cells, and retinal ganglion cells as well as the Müller glia cells (Fig. 2a). The retinal progenitor cells (RPCs) of the optic cup are multipotent and give rise to clones of varying size and fate composition in the adult retina (Fig. 2b). It has been suggested that retinoblastoma can arise from four different retinal cell populations; (1) a retinal stem cell, (2) a proliferating progenitor cell, (3) a newly post-mitotic cell, or (4) a differentiated cell that re-enters the cell cycle (Fig. 2c) [33]. One study indicates that it is precursor cells, and not progenitor cells, that are the cell-of-origin in retinoblastoma [34]. A study in mice indicates that there is a limited time window during development where retinoblastoma can develop, and that in adult 3-week-old mice few retinoblastomas emerged when MYCN was overexpressed in retinas lacking *RB1* [31]. Based on this study it is more likely that the cell-of-origin is a cycling progenitor cell or a newly post-mitotic cell. Another possibility is that the cell-of-origin varies between patients and between tumors within a single patient. In the familial form of retinoblastoma multifocal tumors can be found suggesting that the tumors arise from several different cells, each with the possibility to carry their own defined sets of mutations. The timing of the mutations and the competence state of the cell will then determine the progression and the molecular footprint we use to define the cell type. It is unlikely that non-cycling cells acquire two *RB*1 mutations, suggesting both a difference between the familial and the non-hereditary forms and an increased probability to initiate the cancer from a cycling progenitor. However, further studies are needed in order to elucidate when and from what type of cells the tumor arises.

Studies on human samples indicate that the cell-of-origin is likely a cone photoreceptor [35, 36] whereas studies in mouse models suggest a horizontal cell [37], or a Müller glia cell [38]. At single cell level, retinoblastoma cells have been found to have hybrid expression signatures, co-expressing developmental programs of photoreceptors, horizontal and amacrine cells, as well as retinal progenitor cells [39]. It should be noted that Müller cells and retinal progenitor cells have similar expression profiles, with the glia cell retaining some stem cell like properties for regeneration, which may explain the glial component in retinoblastoma. These conflicting expression patterns may suggest that the cell-of-origin is a multipotent progenitor cell of varying competence providing a heterogenic character to the developing tumor. It may therefore be difficult to pinpoint the cell-of-origin for retinoblastoma. However, as the molecular traits of ganglion cells and bipolar cells are never observed in the tumors, it is important to understand the common traits, the molecular pathways and cellular characteristics, of the cells found in retinoblastomas.

As mentioned, the retinal cells are derived from a common, multipotent progenitor cell [40], and the photoreceptors and horizontal cells often share an immediate progenitor (Fig. 2c) [41, 42]. A common photoreceptor and horizontal cell progenitor was found to express the transcription factor Lim1 [43]. Lim1 expression increases in cells that become horizontal cells [44], whereas Lim1 expression is downregulated in photoreceptors [45]. Lim1 may function as a potential oncogene since increased levels of both Lim1 mRNA and protein was found in human renal cell carcinoma, compared to normal tissue [46]. If Lim1 is a potential oncogene and it is expressed in the immediate common progenitor for photoreceptors and horizontal cells then it may be the progenitor that is the cell-of-origin, potentially explaining the presence of genetic markers for both photoreceptors and horizontal cells in retinoblastoma [35, 37]. It would therefore be of interest to investigate a potential correlation between Lim1, pRb and additional genes such as *MYCN* and *OTX2*. An additional interesting trait is that both photoreceptors and horizontal cells in the zebrafish retina arise from symmetric division of partly differentiated precursors [42]. This suggests that these cells, against common perception, represent neurons that can proliferate in a differentiated state.

### Resistance to apoptosis

A study performed in mouse indicates that retinoblastoma arise from a precursor cell that is naturally death-resistant [34]. Resistance to apoptosis is a hallmark of cancer and increased resistance to apoptosis is a cellular trait that would reduce the number of steps needed for tumor transformation. Cellular stress and excess proliferation result in changes in the phosphorylation of pRb, thereby releasing E2Fs leading to apoptosis [8]. Loss of pRb results in an increased signaling of DNA damage pathways and to increased cell death [47]. In the absence of pRb some retinal cell types, such as ganglion cells, bipolar cells and rod photoreceptors underwent apoptosis while horizontal and amacrine cells did not [34, 48]. There is evidence that the final number of photoreceptors and horizontal cells during development is not regulated by apoptosis, as is the case for the other retinal cell types [49, 50]. In the mature retina, photoreceptor cell death is a common feature in a number of different eye diseases. However, a study performed on a degenerative disease in the dog retina revealed that terminally differentiated photoreceptors have the capacity to proliferate, despite carrying the degenerative mutation [51]. Studies performed on horizontal cells showed that they have the capacity to evade apoptosis and continue dividing in the presence of DNA damage, despite having a functional DNA damage response [52, 53]. The DNA damage response pathway includes activation of the transcription factor and tumor repressor p53, leading to cell cycle arrest [54]. Retinoblastoma cells tend to express wild type levels of p53 [55]. The activity of p53 is regulated by different modulators, such as Zac1 [56], and while overexpression of *Zac1* in the chicken embryonic retina triggered apoptosis, the horizontal cells withstood the effect of p53 activation and continued dividing [57]. The discovery of polyploid horizontal cells, with a replicated genome [58], represents an additional trait that could potentially increase the genomic stability and confer a higher resistance towards cell cycle arrest and/or apoptosis. This suggests that horizontal cells have an atypical regulation or execution of their p53 system, which strengthens the notion that they are less sensitive to signals that regulate cell cycle progression [59]. Combined these results may explain the involvement of photoreceptors and horizontal cells in retinoblastoma.

Although both horizontal cells and photoreceptors may have an intrinsic propensity for neoplastic transformation, due to their suggested inability to be cleared via apoptosis, it is possible that the presence of crosstalk and feedback regulatory loops between *OTX2* and *MYCN* pathways may be one of the components that drives the tumorigenesis in cells with photoreceptor-like molecular signature.

## Heterogeneity in tumor biology and clinical relevance

The genetic and cellular heterogeneities observed in retinoblastoma affect tumor biology, giving rise to both intrapatient and intratumor variance. A better genotype–phenotype correlation may help us to understand the mechanisms behind tumorigenesis as well as identify relevant drug targets.

### Retinoblastoma phenotypes

**Fig. 3:**
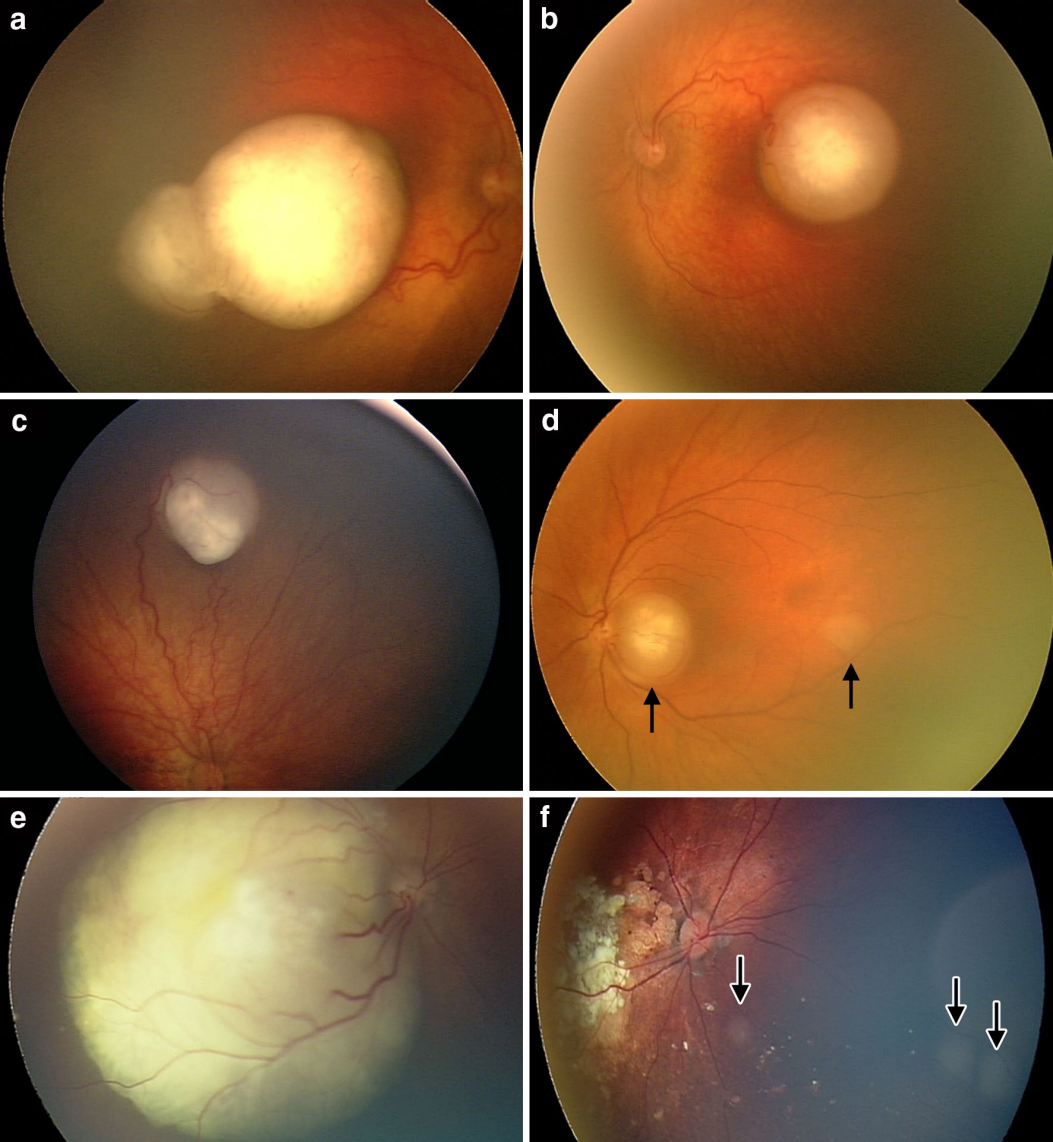
RetCam images of eyes affected by retinoblastoma. **a**, **b** An example of bilateral tumors. Right eye (**a**) presents, with intergrowth between, two initially separate tumors. **c** Unifocal and **d** multifocal tumors. Tumor before (**e**), and after (**f**) treatment with intra-arterial chemotherapy. Note the presence of new multifocal tumors after treatment. Arrows indicate tumors

In addition to having heterogeneity on the genetic and cellular levels, retinoblastoma displays variation in tumor biology and clinical characteristics, which affects both diagnosis and treatment. The tumors can affect one eye (unilateral) or both eyes (bilateral; Fig. 3a, b), and in 5% of children with heritable retinoblastoma it is associated with a midline brain tumor (trilateral/pineoblastoma) [60]. The tumors can be unifocal (Fig. 3c), a single tumor, or multifocal (Fig. 3d), where additional tumors arise from the original clone. Patients with the familial form or de novo germline mutations often develop bilateral early onset tumors that are multifocal, whereas patients with the sporadic form usually develop unilateral tumors that are unifocal [8].

It is at this point not fully clear if multiple foci tumors, which may originate at different time-points, grow and merge to generate tumors of variable origin. Expression analysis of 21 retinoblastomas by Kapatai et al. suggests the existence of two retinoblastoma sub-types: one expressing multiple retinal cell markers and aforementioned recurrent chromosomal alterations typical of retinoblastoma, and another with the characteristics of cone photoreceptors with high metabolic capacity and proliferation [61]. Josephine Dorsman and colleagues explored the nature of this heterogeneity and drew the conclusion that retinoblastoma tumors start as smaller, more differentiated tumors with fewer somatic DNA copy number alterations and progress towards larger, less differentiated, more proliferative and more genomically instable tumors [62].

Families with highly penetrant retinoblastomas often carry mutations that result in early stop codons. These are often nonsense or frameshift mutations within exons resulting in the absence of pRb or loss of its function [63, 64]. On the other hand, in families with low penetrance retinoblastoma it is more common with small in-frame insertions, deletions and splicing mutations [65]. These are mostly missense mutations or mutations in the promoter region that results in lower efficiency or lower levels of the pRb protein. Patients with *RB1* mutations that affect E2F binding, with retained ability to induce differentiation of tumor cells, had low penetrance of retinoblastoma [66]. The carriers in these low-penetrance families develop unilateral tumors, retinomas, or no tumors at all [67]. In two different studies it was shown that a parent-of-origin effect is involved in low penetrance families. In families segregating the c.1981C > T/p.Arg661Trp mutation of *RB1* the probabilities of being unaffected for germline carriers were 90.3 or 32.5% depending on if the mutation was inherited from the maternal or paternal side, respectively [68]. This maternal protective effect was also found in the low penetrance mutation c.607 + 1G > T [69]. Advanced paternal age increases the risk for retinoblastoma suggesting that genomic alterations may increase in aging men [70]. As the results suggest that heterogeneity in *RB1* mutations affect both the penetrance of retinoblastoma and likely tumor progression [66] it stresses the importance of analyzing parents with hereditary forms of retinoblastoma.

### Intrapatient heterogeneity

The most common clinical signs of retinoblastoma are leukocoria (white pupil, 60%) and strabismus (misaligned eyes, 20%) [71]. Upon initial diagnosis of retinoblastoma, rapid identification of whether it is a germline *RB1* mutation, sporadic *RB1* mutation or a RB1^+/+^ MYCN^A^ is necessary for correct treatment, as well as risk assessment for patents and their families [12]. Patients with heritable retinoblastoma or heterozygous carriers have an increased risk of developing secondary tumors such as osteosarcomas, soft tissue sarcomas, and malignant melanomas [72]. They should therefore undergo regular follow-ups throughout their lifetime, and if possible, avoid all radiation (including X-ray, CT scan and external beam) to minimize the lifetime risk of developing secondary cancers. Furthermore, survivors of a bilateral retinoblastoma with an inherited germline retinoblastoma have a slightly higher risk of secondary cancers compared with those with a de novo germline *RB1* mutation [73]. Patients with a familial *RB1* mutation should be offered preimplantation genetic diagnosis to avoid passing on the mutation to the next generation [74].

Children with RB1^+/+^ MYCN^A^ are younger at diagnosis; 4.5 months compared to 24 months and 21.5 months for unilateral sporadic RB1^−/−^ and children with RB1^+/+^ [22]. This variant of retinoblastoma is non-hereditary, has no increased risk for tumors in the other eye or for other cancers throughout life, however they are highly aggressive why enucleation is currently the best treatment.

Histomorphologically, retinoblastoma is one of the “smallround blue cell tumors” (SRCT). Retinoblastoma may histologically be confused with other SRCT tumors such as lymphoma, rhabdomyosarcoma, nephoblastoma (Wilms´ tumor), Ewing´s sarcoma/PNET and desmoplastic small round cell tumor. The retinoblastoma tumor cells form characteristic Flexner-Wintersteiner (FW) rosettes, in which the tumor cells surround a central lumen lined by basement membrane material or Homer-Right rosettes without any lumen [75]. However, RB1^+/+^MYCN^A^ retinoblastomas have distinctive histological features with undifferentiated cells with large prominent, multiple nucleoli, necrosis, apoptosis and little calcification. Data is conflicting regarding the presence of FW rosettes in RB1^+/+^MYCN^A^ tumors [22, 32].

### Intratumoral heterogeneity

Heterogeneity is also present when it comes to the malignancy of the tumors. Retinoma/retinocytoma is a rare, benign variant of retinoblastoma that is also caused by mutations in the *RB1* gene. Studies have shown that retinoma is a precursor of retinoblastoma that may undergo malignant transformation [24, 76]. This data was confirmed in several studies where even retinoma and retinoblastoma was displayed adjacent to each other in the same eye tumor sample [24]. For example, results showed progressive gain of *MYCN* and *E2F3* as well as high expression of p16INK4a and p130 in retinomas compared to a low expression in retinoblastoma [23, 24].

### Treatment strategies

**Fig. 4:**
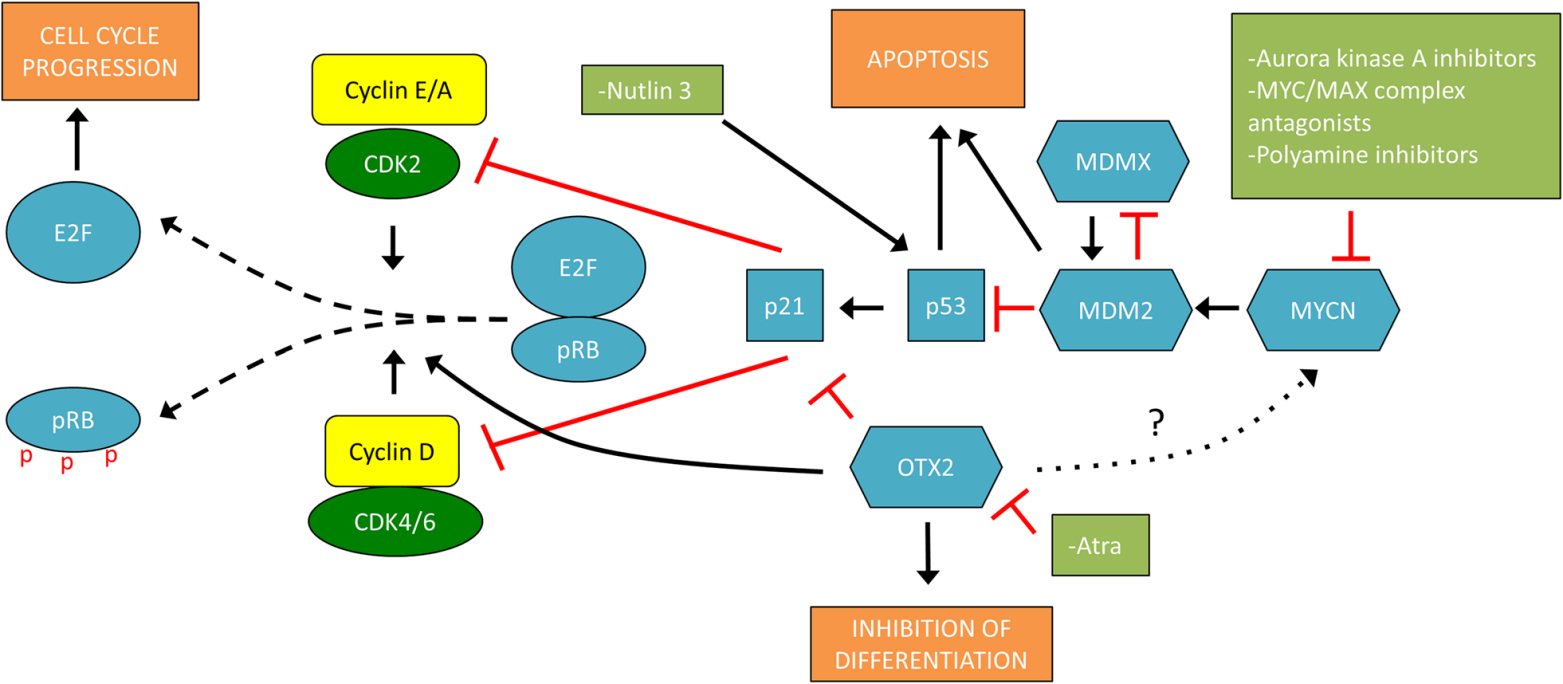
Overview of the molecular pathways involved in regulating the cell cycle, differentiation and apoptosis. Different strategies have been proposed for direct and indirect targeting of p53, MYCN and OTX2 in retinoblastoma. Nutlin-3 has been shown to stabilize p53. Taking inspiration from neuroblastoma treatments, MYCN activity can be blocked by disruption of the MYCN/MAX dimerization complex, or introduction of MYCN protein destabilization via aurora kinase A inhibitors. Pharmacologic inhibition of OTX2 activity via all-trans retinoic acid (ATRA) can increase differentiation and apoptosis and decrease proliferation in cancer cells

A better understanding of the correlation between specific mutations and tumor biology could offer insight for developing more target-specific therapies for patients with retinoblastoma (Fig. 4). The current treatment of retinoblastoma is based on tumor size, number of tumors, tumor stage and localization, presence of vitreous seeding and age of the child. Treatment options are enucleation, focal treatment with cryotherapy, photocoagulation and brachytherapy, often in combination with systemic chemotherapy. Lately, intra-arterial chemotherapy (Fig. 3e, f) and intravitreal injections allow for enhanced treatment outcomes with higher percentage of eye-sparing cases. Adjuvant chemotherapy is utilized for eyes with optic nerve invasion beyond the lamina cribrosa or massive choroidal invasion [77]. Extraocular or orbital disease is treated with radiation and chemotherapy.

A number of epigenetic therapies are found to be effective in multiple cancer types. In the process of finding new targeted therapies for retinoblastoma Kooi et al. aimed to find recurrent genetic alterations subsequent to *RB1* loss that drive tumor progression. They found that single nucleotide variants, insertions and deletions, are very rare beyond *RB1* inactivation and that targeted therapies based on secondary alterations would probably have an effect on only some parts of the tumor in some retinoblastoma patients [30]. Another study by Zhang et al. showed promising results regarding the *SYK* proto-oncogene, which is important for cell survival. Inhibiting *SYK* with a small molecule inhibitor caused degradation of MCL1 and caspase mediated cell death in retinoblastoma cells in vitro and in vivo and may be a drug target for retinoblastoma [78]. It has also been shown that Nutlin-3, a blocker of the MDM4-p53 interaction, could be used as a therapeutic approach [79]. However, it was only the retinoblastoma cell line Weri1, with wild-type p53 and MDM4 amplification, and not a mouse derived p53-deficient cell line that responded to the treatment (Fig. 4).

If the retinoblastoma tumor contains a *MYCN* amplification it may be of interest to target that gene. *MYCN*, together with *c*-*Myc* and *MYCL* are part of the MYC family of oncoproteins. The genes are transcription factors that control the expression of certain cell cycle genes [28] by dimerizing with the MAX protein [80] and binding to the E-box motif [81]. A suggested therapeutic approach to target the MYC oncoproteins is the addition of a MYC/MAX complex antagonist. Such antagonists have been found to compete for binding to the E-box and subsequently repress transcription [82]. It has also been suggested that polyamine inhibitors can be used against MYCN, as a study in where they used two such inhibitors showed a severe growth inhibition and downregulation of *MYCN* [83]. Another therapeutic approach would be to target the kinase aurora A, which stabilizes MYCN and counteracts its degradation [84]. In a recently published paper an aurora kinase A inhibitor was successfully used to reduce MYCN protein levels in a retinoblastoma cell line [31]. Taken together these studies indicate that it may be possible to target MYCN in the MYCN^A^ tumors (Fig. 4).

The homeodomain transcription factor *OTX2* is important for the formation of photoreceptor cells [85]. Aberrant overexpression of *OTX2* has been detected in both retinoblastoma tumors and cell lines, which makes it plausible that there is a link between the photoreceptor-like characteristics in these cancer cells and the proposed cell-of-origin [86–88]. Bunt et al. linked silencing of *OTX2* to downregulation of Cyclin D1 and upregulation of p21 [89]. Knock-down of *OTX2* expression has also been shown to increase the phosphorylation of pRb, which would affect cell proliferation [86]. Pharmacologic inhibition of *OTX2* via all-trans retinoic acid (ATRA) caused increased apoptosis, decreased proliferation and colony formation in retinoblastoma cell lines [86]. High expression of *OTX2* in medulloblastoma together with c-*MYC* and *MYCN* oncogenes, correlates with anaplasticity and unfavorable patient outcome [90]. Studies show that *OTX2* may give stem cell-like properties to tumor cells via epigenetic regulation and transcriptional repression of differentiation markers [89, 90]. Furthermore, *OTX2* has been shown to directly upregulate the expression of c-*MYC* in medulloblastoma via cis-regulatory elements in the *MYC* promoter region [91, 92]. Taking into account that *MYCN* and c-*MYC* are thought to be able to compensate for each other [29], one could speculate that *OTX2* expression in early photoreceptors would mediate an increased cell proliferation in these cell in the case of failed cell cycle exit during tumorigenesis. It is interesting to note that trilateral tumors, the pineoblastoma affecting the light-sensitive pineal gland, involve cells with very similar properties and genetic components as the retinal cells. In fact, there have been cases reported of concurrent trilateral retinoblastoma and medulloblastoma in the same patient [93–95], which additionally supports the theory about the role of *OTX2* as an oncogene in these cancers. This data makes *OTX2* an interesting therapeutic target for retinoblastoma (Fig. 4).

## Current and future platforms for studying retinoblastoma

**Fig. 5:**
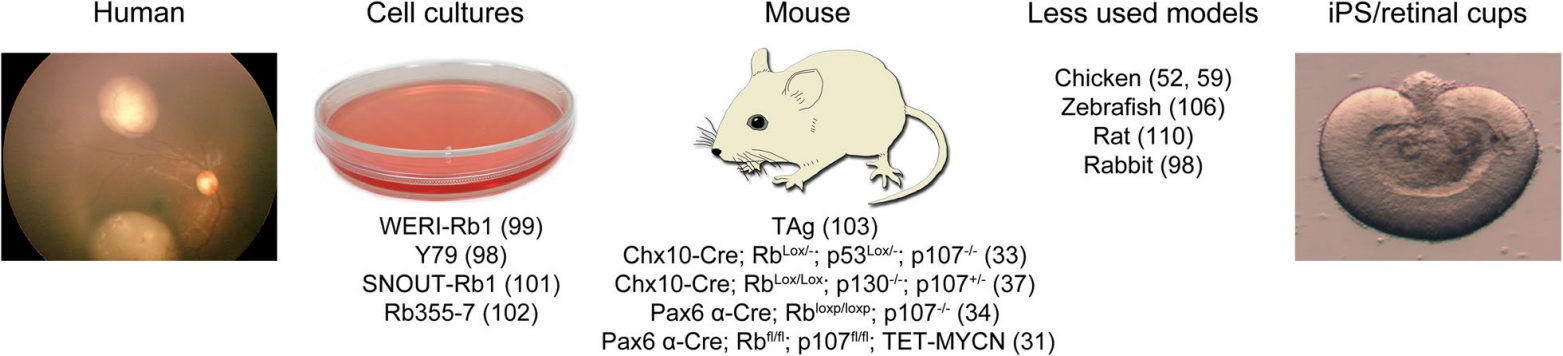
Research model for retinoblastoma. As human tumors are difficult to analyze other in vitro and in vivo models have been developed. There exist several cell lines that have been established from human tumors. Multiple mouse models have also been developed to recapitulate retinoblastoma with a selection presented here. Other, less used models include zebrafish, rat, and rabbit, where primarily transplantation experiments have been performed. Retinal cups from iPS cells have emerged as a promising technique for modeling retinoblastoma that may have a closer resemblance to the initiation and progression of the disease

Over the years, many different approaches have been used to study retinoblastoma (Fig. 5). Sporadic development of retinoblastoma in nonhuman species appears almost nonexistent, although, there has been one documented case in a dog [96]. Therefore, in order to study retinoblastoma formation, cell-of-origin and the underlying causes, one has to rely on in vivo and in vitro models. Each approach has its own advantages and disadvantages and there is substantial variance in phenotype and biology between different models. The over-all goal with a model should be focused on the ability to follow tumor initiation and progression, how metastases are formed, and provide a useful platform for assessing different therapies.

### Human tumors

Since retinoblastoma is a rare pediatric cancer that develops in utero, it is difficult and ethically questionable to study in human embryos. Enucleated eyes from patients provide one source of material to study (Fig. 3). However, because of the advanced stage of the disease at the time of enucleation, possibly following laser- or cytostatic treatment, the obtained tissue makes it often difficult to draw conclusions about the cell-of-origin and understand events that take place early on in the developing retina.

When analyzing the gene expression profiles in 52 fresh retinoblastomas from patients who underwent surgical enucleation prior to anti-cancer treatment, McEvoy et al. observed multiple neuronal differentiation programs in the tumors, not present in normal retina. The authors were unable to identify the cell-of-origin from the properties of the tumor cells, due to presence of altered differentiation pathways during tumorigenesis [97].

### Cell lines

Retinoblastoma cell lines from patients or animal models have also been used. The two most commonly used human retinoblastoma cell lines are Y79 and Weri1. Y79 was isolated from a 2 year old girl with a family history of retinoblastoma [98] while Weri1 comes from a 1-year-old girl with no family history of the disease [99]. Both lines have an *RB1* ^−/−^ genotype with homozygous deletions in *RB1* [22]. Unfortunately, there is data suggesting that both cell lines have undergone significant changes and/or selection in culture that distinguish them from the primary human retinoblastomas [100]. Other human cell lines have also been established and studied, such as SNOUT-Rb1 [101] and Rb355-7 [102]. It is interesting to point out that *MYCN* amplification is often much higher in cell lines than in primary tumors [21, 22]. Long-term cultivation of cell line could reflect the events taking place during metastasis and optic nerve invasion [100]. Primary mouse retinoblastoma cell lines, such as SJmRBL-8, have also been cultured and analyzed [100]. One disadvantage with cell lines is that prolonged in vitro culturing may lead to changes in their genetic background or behavior, and there is also a risk that certain cells are selected based on their high proliferation capacity or whether they grow adherent or non-adherent [100].

### Mouse models

Genetically modified mice present a useful model as tumor development can be studied both during embryonic and post-embryonic development (Fig. 5). Several different mouse models have been used to study retinoblastoma, such as the transgenic model that expresses the oncogenic simian virus 40 T antigen (TAg) under the control of the luteinizing hormone β subunit [103], the Chx10-Cre; Rb^Lox/−^; p53^Lox/−^; p107^−/−^ knockout model [33], the Chx10-Cre; Rb^Lox/Lox^; p130^−/−^; p107^+/−^ knockout model [37], the Pax6 α-Cre; Rb^loxp/loxp^; p107^−/−^ knockout model [34], and the Pax6 α-Cre; Rb^fl/fl;^; p107 ^fl/fl^; TET-MYCN knockout model [31], to mention a few.

Rb1 mutations in mice did not result in tumor formation and it seem as the family member p107 takes the role of Rb1 in the murine retina. Unlike the human developing retina, postnatal mice retinas do not express Rb1 but rather upregulate p107 [104]. The Rb1, p107 double knock-out is embryonically lethal forcing a Cre-activated excision driven by various promoters. The discrepancies in results between studies have been high, which may be explained by the different means of inducing the disruption of *RB1*. The transgenic lines driving the excision of *RB1* affect the timing of the mutation, which in turn affect the progression and competence of tumor cell. There is also the obvious variation caused by the combination of genes that is knocked out.

### Chicken

The chicken embryo provides a system for studies of retinal development and retinal cell type ontogeny that have contributed to the understanding of the properties of the cell-of-origin for retinoblastoma as previously discussed [52, 53, 57–59]. The possibility to target specific retinal cell types including the cell-of-origin for retinoblastoma at defined developmental time points by accessing the embryonic eye in ovo, using chemical or genetic tools [41], gives opportunities to ask questions about the early steps in retinoblastoma carcinogenesis in vivo. Such questions may be related to formation of both retinoma, retinoblastoma and their relationship. The versatile chicken embryo system may also serve as an “incubator” that allows testing mechanistic questions that then may be brought to more elaborate human stem cell derived retinoblastoma models.

### Zebrafish

A study performed in zebrafish showed that individuals carrying a *rb1* gene with a mutation, causing a premature stop codon in exon 20, have a delay in cell cycle exit of the early born retinal ganglion cells leading to optic nerve hypoplasia [105], demonstrating the importance of the gene in proper retinal development. The *rb1* gene has also been inactivated in zebrafish by injection of TALENs [106]. The authors described the presence of tumors that protrude out over the eyes and cause malformations of the skull. It was, however, not clear if they had found any tumors originating specifically from the retina. Heterozygous *rb1* ^+/−^ adults carrying a germline mutation did not develop tumors.

The zebrafish holds great potential to be a useful model for retinoblastoma studies as it has an easily accessible transparent embryo, a rapid retinal development and it allows for the use of a wide variety of techniques whereby the *rb1* gene, or other genes of interest such as *MYCN* and *OTX2*, can be mutated, overexpressed or deleted.

### Orthotopic transplantation models

To study the tumorigenic capacities of cells or to test different treatments, orthotopic transplantations have been performed into a number of different species, such as mouse [100, 107, 108], rabbit [98, 109], rat [110, 111], and zebrafish [112, 113]. The transplantations are performed subretinally, between the retina and pigment epithelium, or intraocularly, into the vitreous body. Intraocular injections are the easiest to perform out of the two; however, the cells will be localized in the vitreous body, and not necessarily in contact with the retina. Subretinal injections will place the cells in direct contact with the retina, and should therefore more closely resemble the human retinoblastoma. For tracing and analysis purposes it is highly advantageous to have the retinoblastoma cells labeled with a traceable compound such as DiI [112], or a genetic marker such as green fluorescent protein (GFP) [113]. One important aspect to consider when performing orthotopical transplantations is that the cells have been cultured in vitro in culture medium for various amount of time. They may therefore no longer fully resemble the cells extracted from the primary tumor. In addition, orthotopical injections lead to an increase in the number of cells, and also a sudden increase in pressure, in the vitreous body, which may affect the eye.

### Stem cells

The fast progress in the field of stem cell research, including differentiation of stem cells into fully laminated three-dimensional retinal cups [114], as well as generation of induced pluripotent stem cells from retinoblastoma patients [115], offer new tools for mimicking the origin and tumorigenesis of retinoblastoma. RB1-null human embryonic stem cells, generated using CRISPR/Cas9, were shown to form large teratomas with characteristics similar to trilateral retinoblastoma tumors and were proposed as a platform for studying mitochondrial phenotyping, in vivo tumorigenesis, and drug screening [116]. Other interesting models may soon become available. For example, an in vitro human retinal organoid system could be used to replicate the genetic and cellular events that normally take place in utero. Previous attempts to generate a similar model [117] failed to induce Rb expression in maturing cone precursors and further efforts are necessary. A platform for initiation of retinoblastoma in a developing human retina in vitro would allow unique freedom to test various temporal settings for the mutagenic events e.g. loss of *RB1* function on different levels or gain of a *MYCN* or *OTX2* amplification at different steps in retinal development. By looking at the behavior of specific retinal cell types following these events in this human retinal cup system it could be possible to narrow down the search for the cell-of-origin. Finally, this platform could act as a more precise human-specific drug-screening platform.

In order to investigate the potential effects on cell-of-origin and tumor heterogeneity based on the timing of the mutational events it is essential to establish reliable models where both temporally controlled knockout and amplification/overexpression of specific genes can be performed. At present, the mouse is the most used animal model to study retinoblastoma and it would be beneficial to establish other reliable models that could confirm and complement these studies. The findings that could be obtained from the combined use of in vivo and in vitro models would permit a better understanding and interpretation of the heterogenic dataset. It would also make it possible to evaluate risk, based on genetic background and heritability, as well as improve prognosis and patient-specific treatment strategies.

## Concluding remarks

Retinoblastoma is a heterogeneous childhood cancer and the study of its genetic, cellular and clinical variance has aided in the understanding of many important mechanisms in cancer biology. Yet, numerous pieces of the puzzle are still missing in the story about how, when and why the perfectly orchestrated machinery of retinogenesis is disturbed following the loss of *RB1* or amplification of *MYCN*. It is particularly interesting why tumors only form in the retina. The transparency of the eye permits unique visualization of retinoblastoma progression. However, due to the high risk of damaging the eye and the spreading of cancer cells, biopsies are never performed. Therefore, characterization of tumors relies on data from enucleated eyes, which are usually in the late stage of the disease, and has possibly undergone aggressive treatment. New emerging cell and animal models are of great importance for identifying the cell-of-origin for retinoblastoma and for mapping of the early events in tumorigenesis. These should be complemented, if possible, by new in vitro human organoid systems that would avoid the species-specific variation and provide a unique opportunity to mimic the events that normally take place in utero.

The current treatment strategies are gradually shifting from standard tumor management, with the goal to save the patient’s life, towards more advanced tailored therapies that could preserve useful vision, and reduce treatment-related additional risks. New molecular targets and drug carriers are currently being explored, as is the genetic mutation—clinical phenotype correlation. Finally, new technology is needed for more accurate diagnosis to detect *RB1* vs. *MYCN* mutations. In all, better understanding of the heterogeneity observed at the molecular, cellular, and tumor level, as well as the model systems used, will pave this new path towards more efficient patient-tailored treatments.

## Abbreviations

RB1: retinoblastoma gene 1
pRb: retinoblastoma protein
MYCN: v-myc avian myelocytomatosis viral related oncogene, neuroblastoma derived
MYCN^A^: MYCN amplification
OTX2: Orthodenticle Homeobox 2
